# In vitro study of the tocolytic effect of oroxylin A from *Scutellaria baicalensis *root

**DOI:** 10.1186/1423-0127-16-27

**Published:** 2009-03-04

**Authors:** Huey-Chuan Shih, Chun-Sen Hsu, Ling-Ling Yang

**Affiliations:** 1 School of Pharmacy, College of Pharmacy, Taipei Medical University, 250 Wu-Hsing Street, Taipei 110, Taiwan; 2 Department of Obstetrics and Gynecology, Taipei Medical University-Wan Fang Hospital, 111 Hsin-Long Road, Section 3, Taipei 116, Taiwan

## Abstract

Scutellariae Radix is one of the well-known tocolytic Chinese herbs. Oroxylin A is isolated from the root of *Scutellaria baicalensis*. The main syndrome of preterm birth is caused by uterus contractions from excitatory factors. Administration of tocolytic agents is a strategy to prevent the occurrence of preterm births. The aim of this study was to investigate the effects of oroxylin A on contractions of uterine strips isolated from non-pregnant female Wistar rats (250~350 g). Contractions of the uterus were induced with acetylcholine (Ach) (1 μM), PGF_2α _(0.1 μM), oxytocin (10^-3 ^U/ml), KCl (56.3 mM), tetraethylammonium (TEA; 1 and 10 mM), 4-aminopyridine (4-AP; 5 mM), glipizide (30 μM), a nitric oxide synthase (NOS) inhibitor (LNNA; 10^-3^M), a β-receptor blocker (propranolol; 10 μM), and a cyclooxygenase inhibitor (indomethacin; 60 μM). The inhibitory effects of the amplitude and frequency of spontaneous contractions by oroxylin A were antagonized with Ach (IC_50 _22.85 μM), PGF_2α _(IC_50_27.28 μM), oxytocin (IC_50 _12.34 μM), TEA; 1 and 10 mM (IC_50 _52.73 and 76.43 μM), 4-AP (IC_50 _67.16 μM), and glipizide (IC_50_27.53 μM), but oroxylin A was not influenced by Ca^2+^-free medium, LNNA, propranolol, or indomethacin. Otherwise, oroxylin A-mediated relaxation of the rat uterus might occur through opening of uterine calcium-dependent potassium channels or adenosine triphosphate potassium channel activation. This suggests that oroxylin A is the tocolytic principle constituent of *Scutellariae Radix*, and oroxylin A may provide a lead compound for new tocolytic drug development in the future.

## Background

After pregnancy, the endocrinology of the body of pregnant women obviously changes, including uterine contraction agonist receptors (such as oxytocin receptor, prostaglandin receptor, β-adrenergic receptor, and corticotrophin releasing hormone receptor), and ion channel proteins which determine the resting membrane potential and excitability of myocytes [1]. Dysfunctional uterine contractions can lead to premature delivery. Spontaneous preterm labo and delivery accounts for approximately one-third of preterm births, which is the predominant cause of prenatal mortality and morbidity. The wide range of tocolytic agents in use is testament to the fact that we still do not have an ideal drug available [2]. Therefore, development of new safe and effective tocolytic agents is an important research topic.

The Chinese herbs, Huang-Chi, *Scutellaria baicalensis*, has been widely used to treat several diseases such as inflammation, hypertension, suppressive dermatitis, diarrhea, and pyrogenic infections [3]. Oroxylin A (5,7-dihydroxy-6-methoxyflavone) is a flavonoid that is an active component isolated from the root of *S. baicalensis*. Several previous reports suggested that oroxylin A is a potential anti-inflammatory agent [4]. It has suppressive effects on superoxide and nitric oxide (NO) generation [5] and an inhibitory effect on diclofenac 4-hydroxylation (CYP2C9) activity [6]. In addition, it inhibits lipopolysaccharide-induced inducible NO synthase (iNOS) and cyclooxygenase (COX)-2 gene expression by suppressing nuclear factor-κB (NF-κB) activation [7,8], and it has also been reported to suppress lymphocyte blastogenesis [9,10]. However, the effect of oroxylin A on the uterus is still unknown. The tocolytic effect of oroxylin A is demonstrated in the present study.

## Materials and methods

### Chemicals

**Figure 1:**
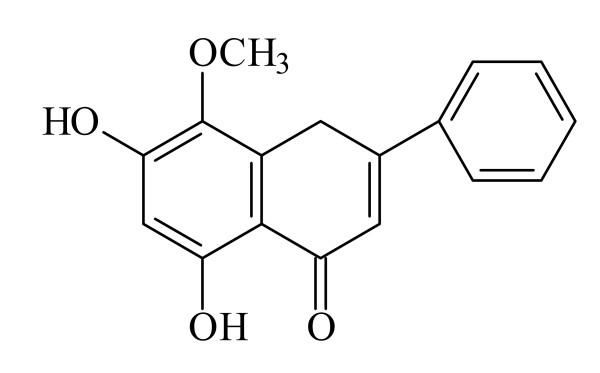
**Oroxylin A isolated from *Scutellaria baicalensis***.

Oxytocin, acetylcholine (Ach), (S)-(-) propranolol hydrochloride, dimethyl sulfoxide (DMSO), papaverine HCl, tetraethylammonium (TEA), 4-aminopyridine (4-AP), glipizide, and N-nitro-L-arginine (LNNA) were obtained from Sigma Chemical(St. Louis, MO). Estradiol benzoate was purchased from China Chemical & Pharmaceutical, (city, Taiwan), and prostaglandine F_2α_(PGF_2α_) from Ono Pharmaceutical (city, Japan). The stock solutions of all drugs were diluted to the desired concentrations with a physiological salt solution. Oroxylin A was isolated from the root of *S. baicalensis *(Labiatae) obtained from Taipei crude drug store and the structure is shown in Fig. 1.

### Extraction and purification of oroxylin A from S. baicalensis

Oroxylin A was extracted from dried *S*. *baicalensis*. In brief, dried *S*. *baicalensis *roots were cut into small pieces, immersed, and extracted with 10-fold v/w acetone twice at room temperature for 2 weeks. After filtration, the residues were reflux-extracted with 4-fold v/w of 50% aqueous ethanol twice for 6 hours. The acetone extracts were subjected to column chromatography on silica gel eluted with CHCl_3 _and CHCl_3_-MeOH, and rechromatographed on silica gel eluted with hexane-acetone to yield oroxylin A. The compound was identified by direct comparison of its electrospray ionization (ESI)-mass, ^1^H- and ^13^C-nuclear magnetic resonance (NMR) spectroscopic data with authentic samples. Purity tests of oroxylin A were performed by high-performance liquid chromatography (HPLC). The HPLC system consisted of a Shimadzu model LC-10AT system (Kyoto, Japan) equipped with a Shimadzu model SIL-9A autoautoinjector and a Shimadzu model SPD-10 A detector(Shimadzu, Kyoto, Japan). Peak areas were calculated with a Shimadzu model C-R8A recorder. A LiChrospher 100 RP-18e reversed-phase column (Merck, Darmstadt, Ger-many) and a LiChrospher 100 RP-18e guard column (Merck) were used. The purity of all compounds exceeded 99.5%.

### Animals

We used non-pregnant female Wistar rats weighing 250~350 g purchased from the Center of Experimental Animals, National Taiwan University, Taipei, Taiwan. All experiments were performed in accordance with guidelines for animal experiments of Taipei Medical University and the guiding principles for the care and use of laboratory animals approved by the Chinese Society of Laboratory Animal Sciences, Taiwan. All efforts were made to minimize animal suffering and to reduce the number of animals used.

### Isolation of rat uteri and contractility measurements in vitro

Animals were sacrificed by decapitation, and the uterine horns were excised and cut into two halves (1 cm). The uteri were immediately removed and placed in a flask containing Locke solution of the following composition (in mmol/L): NaCl 154, KCl 5.63, NaHCO_3 _1.79, CaCl_2_·2H_2_O 2.55, and glucose 5.55. Experiments commenced within 5 min of removal of tissue samples. Preparations were placed in isolated organ baths, incubated in Locke's solution at 37 ± 1°C and bubbled with gas (95% O_2_, 5% CO_2_). The preload was 1 g, and the equilibration period was no less than 45 min [11]. The responses induced were expressed as a percentage of the maximum relaxation by papaverine (PPV; 10^-3 ^M) which was added at the end of each experiment. Contractions were recorded by force displacement transducers (Kent Scientific Corporation, USA) using MP100 workstation software (Biopac Systems, Inc, USA) on a PC.

### Effect of oroxylin A on agonist-induced uterine contractions

A uterine strip was incubated in the Locke solution and equilibrated for 30 min. Contractions were induced by Ach (1 μM bath concentration), PGF_2α _(0.1 μM bath concentration), and oxytocin (10^-3 ^U/ml bath concentration). Oroxylin A was added to the incubation solution and allowed to react for 10 min before a more-concentrated agent was added. Concentration-response curves of the prescription (1~100 μm) were plotted against the phasic response to Ach, PGF_2α_, and oxytocin. Control experiments were performed with the vehicle (0.1% DMSO).

### Effect of oroxylin A on oxytocin-induced Ca^2+^-free uterine contractions

Oxytocin-induced contractions in Ca^2+^-free medium: A uterine horn was equilibrated for 1 h in Ringer-Locke solution. The solution was then replaced with Ca^2+^-free solution containing 3 mM EDTA, and incubation continued for 50 min. Subsequently, the solution was replaced by a Ca^2+^-free solution containing 1 mM EDTA, and the uterus was incubated for an additional 20~30 min. A sustained contractile response to oxytocin (10^-3 ^U/ml) was obtained and cumulative concentrations of oroxylin A were added.

### Effect of oroxylin A on uterine contractions induced by K^+ ^depolarization

The organ was immersed in Jalon-Ringer solution and equilibrated for 20 min. This solution was replaced by a depolarizing solution (KCl 56.3 mM) that caused a rapid contraction, followed by slight relaxation and a prolonged contraction plateau. When the plateau was reached, cumulative concentrations of oroxylin A (1~100 μM) were administered, and concentration-related relaxations were observed.

### Investigations on the involvement of potassium (K^+^) channels

In order to test the involvement of potassium channels in the mechanism of action of oroxylin A, TEA (1 and 10 mM), 4-AP (a K blocker; 5 mM) and glipizide (K_ATP_, 30 μM) were applied to the uterine strips 15 min prior to pre-contraction by oxytocin (10^-3 ^U/ml). The cumulative concentration-response curves of oroxylin A were then constructed and compared with those obtained with uterine strips that had not been treated with these inhibitors.

### Effects of a COX antagonist, NOS inhibitor, and β-adrenergic antagonist on the relaxing effect

The uterine rings were induced by oxytocin and then incubated for 15 min with indomethacin (a COX antagonist, 60 μM), L-NAME (an NOS inhibitor, 100 μM) and propranolol (a β-adrenoceptor antagonist, 10 μM). Oroxylin A to be tested for antagonist activity was then added to the bath and left in contact with the tissue for 10 min.

### Statistical analysis

The results are expressed as the mean ± SE of several preparations (*n*) from different animals. Agonists were added until the steady, largest amplitudes were defined as the maximal contractions. In accordance with the dose-response curves, the concentration of oroxylin A producing 50% of the maximal contraction (IC_50 _value) was estimated. Relaxation was expressed as a percentage of the maximal contraction obtained by additing the agonist. Statistical significance of differences between groups was assessed using Student's *t*-test for unpaired data. *p *values of < 0.05 were considered significantly different.

## Results

### Effects of oroxylin A on spontaneous contractions of the rat uterus

Application of oroxylin A, in a cumulative fashion, inhibited spontaneous contractions of the rat uterus in a concentration-dependent manner, whereas the addition of vehicle alone (0.1% DMSO) had no effect. The amplitude of spontaneous uterine contractions significantly decreased with 43.38 μm oroxylin A compared to the control.

### Effects of oroxylin A on uterine contractions induced by agonists

**Figure 2:**
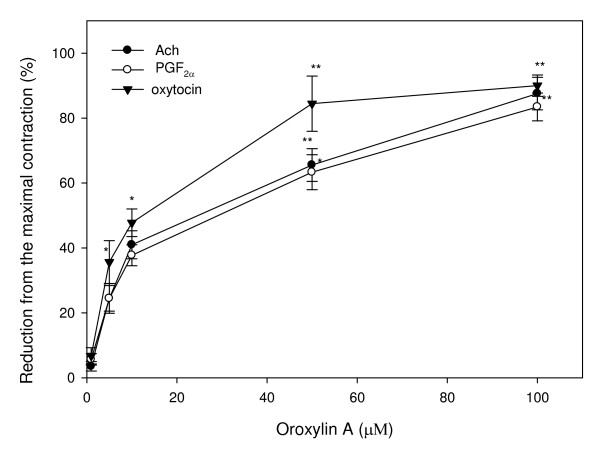
**Effects of oroxylin A on contractions of rat uterus induced by oxytocin (10^-3 ^U/ml), PGF_2α _(0.1 μM), and Ach (1 μM)**. Vertical bars represent the SEM (*n *= 4). *p < 0.05, **p < 0.01.

Agonists, including PGF_2α_, oxytocin, and Ach, can induce intense uterine contractions. We then examined the effects of oroxylin A on uterine contractions induced by these agonists. Oxytocin (10^-3 ^U/ml), PGF_2α _(0.1 μM) and Ach (1 μM) each significantly stimulated contractility. The addition of oroxylin A inhibited uterine contractions induced by three agonists. The inhibition by oroxylin A increased with the concentrations added. The IC_50 _values of oroxylin A on Ach, PGF_2α_, and oxytocin-induced contractions were 22.85 μM, 27.28 μM, and 12.34 μM, respectively. This indicates that oroxylin A was more sensitive in inhibiting oxytocin-induced uterine contractions (Fig. 2).

### Effect of oroxylin A on oxytocin-induced uterine contractions in the Ca^2+^-free solution

**Figure 3:**
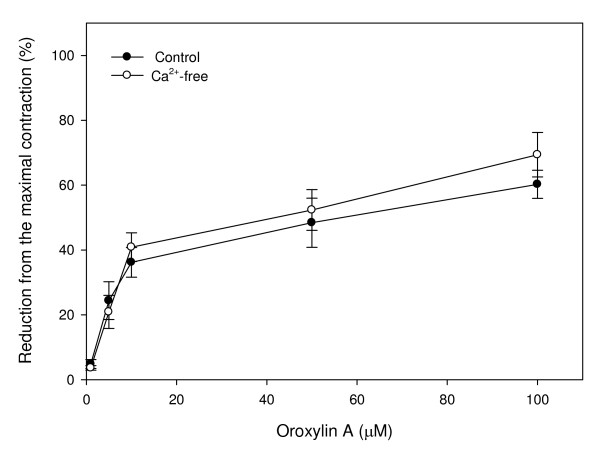
**Effects of oroxylin A on contractions of rat uterus induced by oxytocin (10^-3 ^U/ml) in a Ca^2+^-free solution**. Vertical bars represent the SEM (*n *= 4).

The effect of the oroxylin A on oxytocin (10^-3 ^U/ml)-induced calcium release from smooth muscle in a solution without calcium on uterine contractions is shown in oxytocin (10^-3 ^U/ml) had no inhibitory effect on calcium release from smooth muscle. It had an inducing effect, but there was no statistical significance (Fig. 3).

### Effect of oroxylin A on uterine contractions induced by K^+ ^depolarization

**Figure 4:**
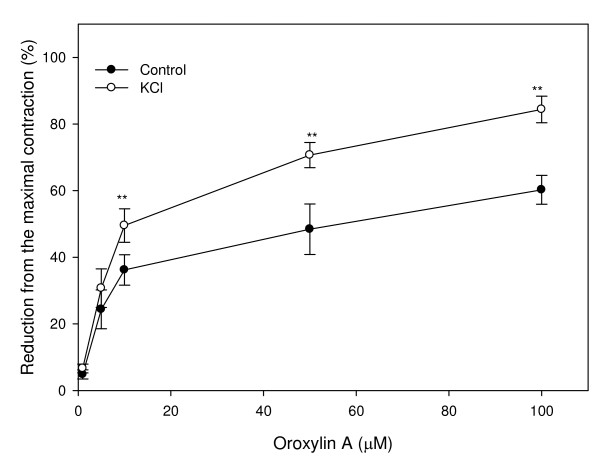
**Effects of oroxylin A on tonic contractions of rat uterus induced by KCl (56.3 mM)**. Vertical bars represent the SEM (*n *= 4). *p < 0.05, **p < 0.01.

The effect of oroxylin A on a high concentration of potassium (KCl 56.3 mM)-induced uterine contractions was examined. Oroxylin A had inhibitory effects on uterine contractions induced by high potassium, and this also showed a dose-response relationship. The IC_50 _of the oroxylin A at 56.3 mM KCl was 12.58 μM. This indicates that alterationsin the of membrane potential are involved in the process of oroxylin A-induced uterine relaxation (Fig. 4).

### Effects of the potassium channel blockers, TEA, 4-AP, and glipizide on oroxylin A induced relaxation in uterine smooth muscle

**Figure 5:**
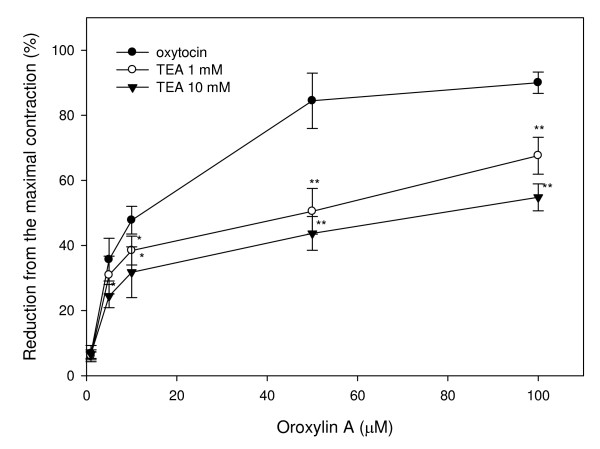
**Effects of the potassium channel inhibitor, tetraethylammonium (TEA; 1 and 10 mM), on oroxylin A-induced uterine relaxation**. Vertical bars represent the SEM (*n *= 4). *p < 0.05, **p < 0.01.

**Figure 6:**
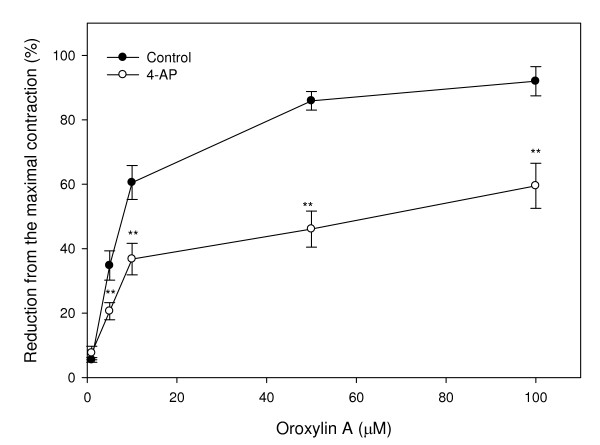
**Effects of the potassium channel inhibitor, 4-aminopyridine (4-AP), on oroxylin A-induced uterine relaxation**. Vertical bars represent the SEM (*n *= 4). *p < 0.05, **p < 0.01.

**Figure 7:**
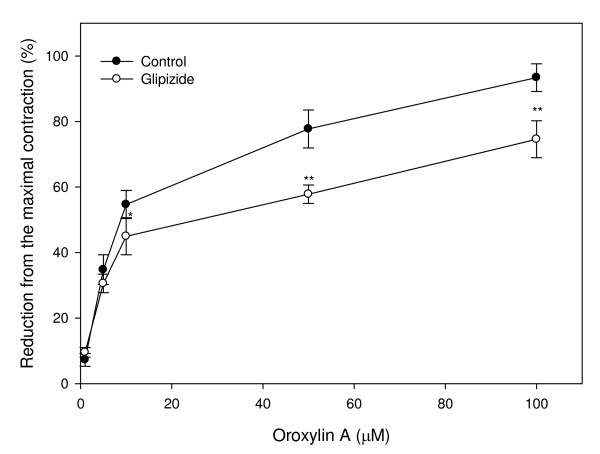
**Effects of the potassium channel inhibitor, glipizide, on oroxylin A-induced uterine relaxation**. Vertical bars represent the SEM (*n *= 4). *p < 0.05, **p < 0.01.

Preincubation of uterine rings from rats with the nonspecific potassium channel inhibitor, TEA (1 and 10 mM), shifted the concentration-response curve of oroxylin A and attenuated the relaxation activity induced by oroxylin A (Fig. 5). Concentrations causing 50% of the inhibition concentration were 52.73 μM and 76.43 μM in the presence of 1 and 10 mM TEA, respectively. The result indicates that relaxation of oroxylin A is inhibited by TEA. In the same part of the experiment, pretreatment of the uterine muscle with other potassium channel inhibitors, including 4-AP and glipizide, showed significant inhibition of oroxylin A-induced relaxation with IC_50 _values of 67.16 μM and 27.53 μM, respectively (Figs. 6, 7). Upon comparison with the results of these potassium channel antagonists, TEA appeared more effective than glipizide in attenuating of oroxylin A-induced uterine relaxation. Therefore, activation of potassium channels in the myometrium including Ca^2+^-dependent or ATP-dependent potassium channels is involved in oroxylin A-induced uterine relaxation.

### β-Adrenergic receptor, COX, and NO were not involved in oroxylin A-induced relaxation

**Figure 8:**
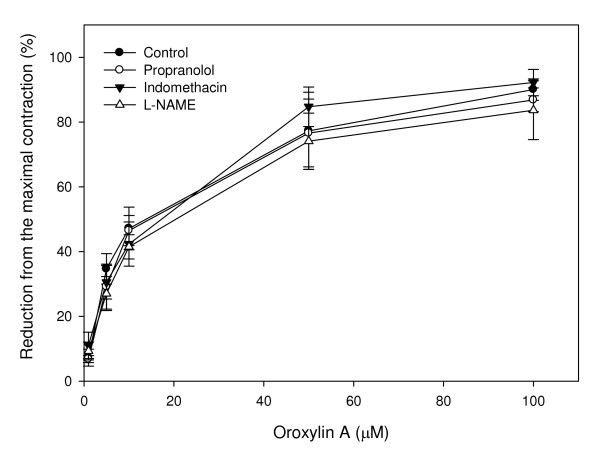
**Effect of the propranolol (β-receptor blocker), indomethacin (cyclooxygenase inhibitor) and L-NAME (NOS inhibitor) on oroxylin A-induced uterine relaxation**. Vertical bars represent the SEM (*n *= 4).

In order to demonstrate the mechanism of oroxylin A-induced uterine relaxation, pharmacological studies were performed to study the roles that β-receptor, prostaglandin, and NO play in oroxylin A-induced uterine relaxation. A β-receptor blocker, propranolol, and a COX inhibitor, indomethacin, and an NOS inhibitor, LNNA, were added to the reaction followed by oroxylin A treatment. The results revealed that propranolol, indomethacin, and LNNA showed no obvious effects on oroxylin A-induced relaxation in oxytocin-precontracted uterine muscle rings. We propose that activation of COX activity, NO production, and the β-receptor might not be essential in the process of oroxylin A-induced relaxation (Fig. 8).

## Discussion

The results of the present study demonstrate that oroxylin A, one of the major components of the Chinese herb, *S. baicalensis*, exerted significant relaxative effects on spontaneous and agonist-induced uterine contractions. The relaxation activity of oroxylin A was attenuated in KCl-contracted uteri and blocked by the addition of TEA and 4-AP to the reaction; however netther L-NNA, propanolol, nor indomethacin showed any effect on oroxylin A-induced relaxation. These data indicate that potassium channel activation is involved in oroxylin A-induced relaxation. These results provide more scientific evidence to support the traditional tocolytic effect of *S. baicalensis *and demonstrate that oroxylin A might be its active component, which deserves further clinical studies.

Cytoplasmic Ca^2+ ^plays an important role in modulating uterine contractions [12]. Previous studies demonstrated that cytoplasmic Ca^2+ ^can be regulated by intracellular and extracellular Ca^2+ ^pools, and agents inducing influx of Ca^2+ ^from extracellular spaces or increasing intracellular Ca^2+ ^by ER release cause uterine contractions. Those studies indicated that Ca^2+ ^is a critical mediator of uterine contractions. In this study, oroxylin A showed a similar relaxation pattern with or without Ca^2+ ^in the reaction. These data indicate that Ca^2+ ^ion might not be an essential factor in modulating oroxylin A-induced relaxation.

At lease three types of potassium ion currents have been described in rat myometrium: a fast transient current and two calcium-dependent noninactivating currents [13]. A single-channel recording experiment revealed that large-conductance calcium-dependent potassium channels appear in the myometrium of both pregnant and non-pregnant [14], and their activation results in cell hyperpolarization and suppression of the concentration of intracellular Ca^2+^. Therefore, a potassium channel opener is a strong uterine relaxant.

Potassium channels are composed of diverse groups of membrane channels including a voltage-dependent potassium channel, a large-conductance Ca^2+^-dependent potassium channel, and a small-conductance potassium channel. Opening of potassium channels causes hyperpolarization of the plasma membrane by increasing potassium conductance and reduces cell excitability by shifting the membrane potential away from the threshold for excitation [15]. To block potassium channels, we used two nonselective potassium channel blockers, TEA, 4-AP, and a selective ATP-dependent potassium channel blocker, glipizide [16,17]. Oroxylin A-induced relaxation of the uterus was completely blocked by TEA, 4-AP, and glipizide. These data indicate activation of potassium channels in the myometrium including Ca^2+^-dependent or ATP-dependent potassium channels is involved in oroxylin A-induced uterine relaxation.

NO is a unique and ubiquitous biological second messenger, and several studies have demonstrated that NO is an important regulatory molecule in uterine contractions during pregnancy. The action of NO in the uterus is believed to consist of maintaining myometrial quiescence during the period of pregnancy, and a decreased amount of NO was seen in uterine smooth muscle in the period near labor and delivery [18,19]. Therefore, NO is an essential factor in the process of pregnancy. In this study, the NOS inhibitor, LNNA, did not suppress the relaxation activity of oroxylin A in oxytocin-precontracted uterine muscle. This result indicates that modulation of NO production might not be involved in oroxylin A-induced uterine relaxation.

Prostaglandins have been reported to be mediators of the regulation of the contraction status during pregnancy and parturition. Prostaglandin synthesis is controlled by two rate-limiting enzymes, phospholipase A_2 _and COX. Two distinct isoforms of COX, COX-1 and COX-2, have been identified. Previous reports demonstrated that inhibition of COX was able to suppress abnormal contractions in the uterus. However, Araslan A et al. demonstrated that inhibition of COX by indomethacin caused a risk of neonatal cerebral hemorrhage and necrotizing enterocolitis [20]. Therefore, the role COX plays in uterus is controversial. In the present study, Indomethacin showed no effect on oroxylin A-induced relaxation. This suggests that COX is not involved in oroxylin A-induced relaxation.

Although oroxylin A will most likely never be used clinically to achieve tocolysis, the data presented here clearly demonstrate that oroxylin A is able to relax pregnant rat myometrium through a mechanism involving activation of potassium channels. The results showed that TEA, 4-AP, and glipizide effectively blocked oroxylin A-induced uterine relaxation. This study demonstrates that the tocolytic effect might be attributable to the opening of uterine calcium-dependent potassium channels or action on ATP-dependent potassium channels. These results indicate that the examined plant possesses direct uterine relaxation activity which can justify oroxylin A usage in traditional herbal remedies during prematurity. This preliminary study has shown that plant extracts should now be subjected to procedures to characterize the pharmacologically active principle, with a view to identifying a novel target site through which to achieve tocolysis.

## Competing interests

The authors declare that they have no competing interests.

## Authors' contributions

HCS designed and performed the experiments, analyzed the data, and drafted the manuscript. CSH co-designed the experiments and participated in discussion of the experimental results. LLY conceived the study, coordinated the implementation of the study, and revised the manuscript.
